# Complete Genome Sequences of *Klebsiella michiganensis* and *Citrobacter farmeri*, KPC-2-Producers Serially Isolated from a Single Patient

**DOI:** 10.3390/antibiotics10111408

**Published:** 2021-11-18

**Authors:** Jehane Y. Abed, Maxime Déraspe, Ève Bérubé, Matthew D’Iorio, Ken Dewar, Maurice Boissinot, Jacques Corbeil, Michel G. Bergeron, Paul H. Roy

**Affiliations:** 1 Centre de Recherche en Infectiologie, Centre de Recherche du CHU de Québec, Université Laval, 2705 boul. Laurier, Suite R-0709, Québec, QC G1V 4G2, Canada; Jehane.Abed@crchudequebec.ulaval.ca (J.Y.A.); maxime@deraspe.net (M.D.); eve.berube@crchudequebec.ulaval.ca (È.B.); maurice.boissinot@crchudequebec.ulaval.ca (M.B.); jacques.corbeil@crchudequebec.ulaval.ca (J.C.); Michel.G.Bergeron@crchudequebec.ulaval.ca (M.G.B.); 2 Département de Microbiologie et Immunologie, Pavillon Vandry, Université Laval, Québec, QC G1V 0A6, Canada; 3 McGill Genome Centre, 740 Avenue Docteur-Penfield, Montréal, QC H3A 0G1, Canada; matt.diorio@mail.mcgill.ca; 4 Department of Human Genetics, McGill University, 3640 rue University, Rm 2/38F, Montréal, QC H3A 0C7, Canada; ken.dewar@mcgill.ca; 5 McGill Centre for Microbiome Research, 3605 de la Montagne, Montréal, QC H3G 2M1, Canada; 6 Département de Médecine Moléculaire, Pavillon Vandry, Université Laval, Québec, QC G1V 0A6, Canada; 7 Département de Biochimie, de Microbiologie et de Bio-Informatique, Pavillon Vachon, Université Laval, Québec, QC G1V 0A6, Canada

**Keywords:** *Klebsiella michiganensis*, *Citrobacter farmeri*, KPC-2, carbapenemase, plasmid, transposon

## Abstract

Carbapenemase-producing *Enterobacterales,* including KPC-2 producers, have become a major clinical problem. During an outbreak in Quebec City, Canada, KPC-2-producing *Klebsiella michiganensis* and *Citrobacter farmeri* were isolated from a patient six weeks apart. We determined their complete genome sequences. Both isolates carried nearly identical IncN2 plasmids with *bla*KPC-2 on a Tn*4401b* element. Both strains also carried IncP1 plasmids, but that of *C. farmeri* did not carry a Beta-lactamase gene, whereas that of *K. michiganensis* carried a second copy of *bla*_KPC-2_ on Tn*4401b*. These results suggest recent plasmid transfer between the two species and a recent transposition event.

## 1. Introduction

Carbapenemase-producing enterobacteria (CRE) have become a major problem throughout the world. The most frequently found carbapenemases are the class A KPC, class D OXA-48 and its variants, and metallo-Beta-lactamases like IMP, VIM, and NDM [1]. Each carbapenemase has a distinct epidemiology. KPC carbapenemases are plasmid-mediated and often found on a Tn*4401b* transposon [2,3]. KPC has spread throughout the United States and into Canada, with outbreaks in Toronto, Montreal, and Quebec City [4,5]. We obtained two strains identified as KPC producers from the Hôtel-Dieu hospital in Quebec City, where five KPC-producing strains were isolated in 2017 from four patients on four different floors. These two strains were anal swab isolates that colonized but did not infect the patient, a 69-year-old male. The first isolate was identified as *Klebsiella oxytoca* (later reclassified as *K. michiganensis*), and the second, isolated six weeks later, was identified as *Citrobacter farmeri*. We report here the sequences of the chromosomes, the four plasmids of *C. farmeri*, and the two plasmids of *K. michiganensis*, which are very similar to two of the plasmids of *C. farmeri*, except that both *K. michiganensis* plasmids carry Tn*4401b* and encode *bla*_KPC-2_, whereas only one of the *C. farmeri* plasmids does.

## 2. Results

### 2.1. Genomic Sequencing

The complete genome sequences of *Klebsiella michiganensis* CCRI-24235 and *Citrobacter farmeri* CCRI-24236 were determined by PacBio and polished with Illumina to resolve homopolymer undercounts (see Materials and Methods). The two chromosomes, two plasmids from *K. michiganensis* and four plasmids from *C. farmeri,* were confirmed as being circular by trimming the terminal duplication of the linear assemblies.

### 2.2. Chromosomes

**Figure 1:**
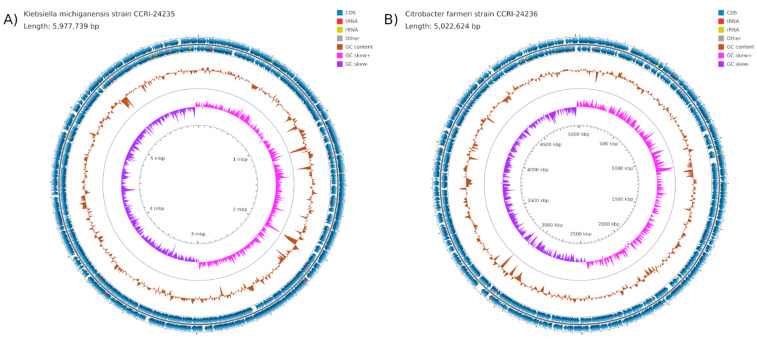
Map of chromosomes of *K. pneumoniae* CCRI-24235 (**A**) and *C. farmeri* 24236 (**B**). The scales are indicated on the innermost circles. The second circles are G+C skew in pink (+) and purple (−), and circles 3 show G+C content (deviation from the average) in brown (+, outward and −, inward). The next two circles illustrate positions of CDSs in minus (circle 4) and plus (circle 5) strands in dark blue.

The chromosome of *K. michiganensis* CCRI-24235 (Figure 1A) was 5,977,739 nt in length. The genome of greatest similarity was that of *K. michiganensis* E718 (CP003683) [6], with an average nucleotide identity (ANI) of 99.49%. The chromosome of *C. farmeri* (Figure 1B) was 5,022,624 nt in length, and among the complete genomes, it was most similar to the *C. farmeri* strain AUSDM00008141 (CP022695) [7] with an ANI of 98.86%. It was also very similar to whole genome shotgun (wgs) genomes of *C. farmeri* 1001216B_150713_F2 and CB00091 (WGS Projects JADMON and JADVHI, respectively) with ANIs of 99.05%. No acquired resistance genes were found in the two chromosomes.

### 2.3. Plasmids pCCRI24235-1 and pCCRI24236-2

**Figure 2:**
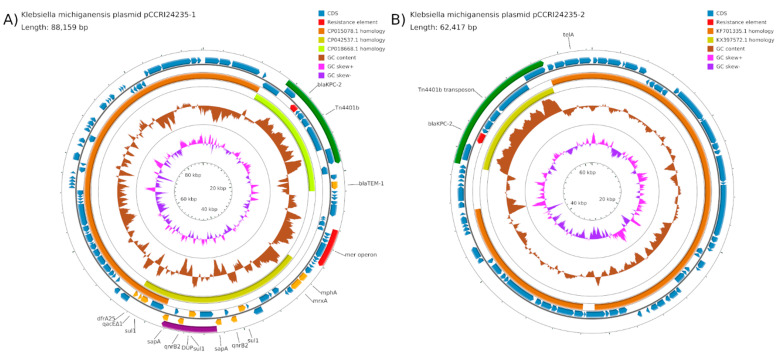
(**A**) Map of plasmid pCCRI24235-1. The scales are indicated on the innermost circles. The second circles are G+C skew in pink (+) and purple (−), and circles 3 show G+C content (deviation from the average) in brown (+, outward and −, inward). The next two circles illustrate regions homologous to related plasmids. The following two circles show positions of CDSs in minus (circle 4) and plus (circle 5) strands in dark blue. The *bla*_KPC-2_ gene is indicated in red. Transposon Tn*4401b* is indicated by a green arc. The segment absent in pCCRI24236-2 (DUP) is indicated by a purple arc. (**B**) Map of plasmid pCCRI24235-2. Transposon Tn*4401b*, indicated in green, is absent in pCCRI24236-3.

Plasmid pCCRI24235-1 from *K. michiganensis* was 88,159 nucleotides in length. Plasmid pCCRI24236-2 from *C. farmeri* was 82,438 nt in length. The two plasmids were identical except for a sequence duplication of the insertion sequence IS*CR1* and an adjacent region, 5721 nt in length. They belong to IncN2 and are very similar to pEC448_OXA-163 from *Escherichia coli* (CP015078; brown arc in Figure 2A). The plasmids contained a Tn*4401b* transposon 10,006 nt in length, encoding a *bla*_KPC-2_ gene. Figure 2A is a map of pCCRI24235-1 and shows the transposon, the *bla*_KPC-2_ gene, and the duplication absent from pCCRI24236-2. In addition to the *bla*_KPC-2_ gene, these plasmids had resistance genes *bla*_TEM-1_, *mphA, mefA, sul1, qnrB2, sapA, qacEdelta1, dfrA25*, and a mercury resistance operon. The integron region is very similar to pE51_003 from *E.* coli (CP042537; dark yellow arc in Figure 2A), while the region containing the *bla*_KPC-2_ and *bla*_KTEM-1_ genes is very similar to pKPC_CAV1042-44 from *K. pneumoniae* (CP018668; light yellow arc in Figure 2A).

### 2.4. Plasmids pCCRI24235-2 and pCCRI24236-3

Plasmid pCCRI24235-2 from *K. michiganensis* was 62,417 nucleotides in length. Plasmid pCCRI24236-3 from *C. farmeri* was 52,406 nt in length. The two plasmids were identical except that the former contained a Tn*4401b* transposon identical to that of pCCRI24235-1 and pCCRI24236-2, and encoding the *bla*_KPC-2_ gene, while the latter lacked the transposon. They belong to a new clade of IncP1 (see Discussion) and are very similar to the *E. coli* plasmid pHS102707 (KF701335; brown arc in Figure 2B) [8]. Figure 2B is a map of pCCRI24235-2 showing the transposon. Plasmids pCCRI24235-2 and pCCRI24236-3 had no other resistance genes except for the tellurium resistance gene *telA*.

### 2.5. Plasmids pCCRI24236-1 and pCCRI24236-4

**Figure 3:**
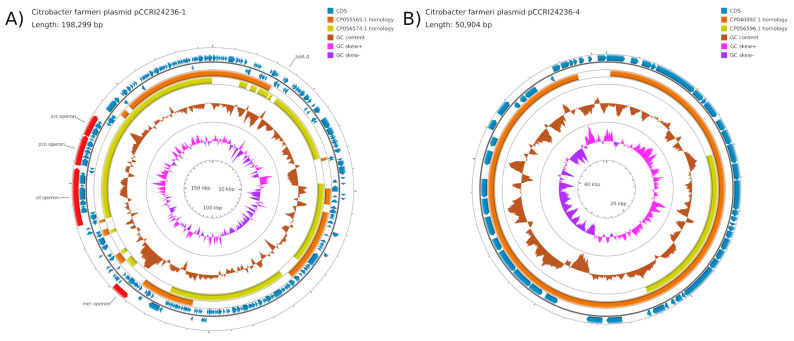
Map of plasmids pCCRI24236-1 (**A**) and pCCRI24236-4 (**B**). The scales are indicated on the innermost circles. The second circles are G+C skew in pink (+) and purple (−), and circles 3 show G+C content (deviation from the average) in brown (+, outward and −, inward). The next two circles illustrate regions homologous to related plasmids. The following two circles show positions of CDSs in minus (circle 6) and plus (circle 7) strands in dark blue.

Plasmid pCCRI24236-1 from *C. farmeri* was 198,299 nucleotides in length. It was identified by PlasmidFinder (see Materials and Methods) as belonging to a novel unknown incompatibility group on the basis of similarity of its *repB* gene to that of pKPC-CAV1321-244 (CP011611), and the whole sequence is closest to *C. freundii* plasmids pRHBSTW-00153-2 (CP055565) and pRHBSTW-00370_2 (CP056574). Figure 3A shows a map of pCCRI24236-1, which had a variety of heavy metal resistance genes, including a copper resistance operon *pcoABCDRSE*. However, tellurium resistance gene *telA* (interrupted by IS*Ecl1*), copper/silver resistance gene *silE* (interrupted by IS*1*), and an arsenical pump-driving ATPase-encoding gene (N-terminal truncated) were pseudogenes.

Plasmid pCCRI24236-4 from *C. farmeri* (Figure 3B) was 50,904 nucleotides in length, belonged to the IncX5 incompatibility group, and was very similar to *Escherichia coli* plasmid pEc1677 (MG516910) [9]. No resistance genes were found.

## 3. Discussion

The two strains, *K. michiganensis* CCRI-24235 and *C. farmeri* CCRI-24236, share a pair of very similar plasmids, with two additional plasmids in the *C. farmeri* strain. The plasmids pCCRI24235-1 and pCCRI24236-2 are IncN plasmids with *bla*KPC-2 on Tn*4401b*, a context first found in KPC-producing isolates in the 1990s [10] that is still very common. Tn*4401* contains *bla*_KPC-2_, a transposase and resolvase, and insertion sequences IS*Kpn6* and IS*Kpn7* [11]. A variety of other elements, collectively called NTEKPC, encode *bla*_KPC_ [12]. Plasmids pCCRI24235-1 and pCCRI24236-2 are identical except for a 5.7-kb duplication of IS*CR1* and adjacent genes in the former. Plasmid pCCRI24235-1 may have evolved from pCCRI24236-2 by a one-ended transposition event mediated by the IS*CR1* transposase [13]. IS*CR1* is found in several integrons downstream of the *sul1* sulfonamide resistance genes [14], and a promoter downstream of the transposase is involved in the expression of downstream resistance genes [15]. In our plasmids, the downstream *sapA* gene is in the opposite orientation. IncP1 plasmids pCCRI24236-3 and pCCRI24235-2 are identical except for the presence of Tn*4401b* in the latter. The location of Tn*4401b* in pCCRI24235-2 represents a novel and unique target. The transposition of Tn*4401b* is likely to have taken place in *K. michiganensis* from pCCRI23235-1 into a plasmid otherwise identical to the *C. farmeri* plasmid pCCRI24236-3, resulting in a second copy of *bla*_KPC-2_ in the *K. michiganensis* strain. The quasi-identity of the two plasmid pairs suggests the possibility of their transfer by conjugation between the two species, although indirect transfer via a third species cannot be ruled out. Plasmid transfer may be between-patient or within-patient events [16], but there were too few isolates from the hospital in 2017 to elucidate the series of events.

Although pCCRI24235-2 and pCCRI24236-3 belong to IncP1, they are in a novel clade that includes pHS102707 (KF701335) [8], pHNFP671 (KP324830), and pMCR1511 (KX377410) [17]. While plasmids of most clades of IncP-1 can be found in *Pseudomonas aeruginosa,* plasmids of this novel clade have not. Plasmid pCCRI24236-1 from *C. farmeri* belongs to a novel unknown incompatibility group; similar plasmids are found in Enterobacteriaceae but not in *Pseudomonas*.

Our results add to the small number of *C. farmeri* complete genomes and show that this species is a factor in KPC dissemination. KPC-producing CRE are still clinically important, although, unlike MBL, they are usually sensitive to certain Beta-lactam/Beta-lactamase inhibitors such as ceftazidime/avibactam. However, various new mechanisms of KPC-mediated ceftazidime/avibactam resistance have been reported [1].

## 4. Materials and Methods

Strains *K. michiganensis* CCRI-24235 and *C. farmeri* CCRI-24236 were obtained from the microbiology laboratory at Hôtel-Dieu de Québec hospital, where they had been isolated from anal swabs of a patient, six weeks apart in 2017, during an outbreak of KPC-2 producers in Québec City. DNA was prepared according to the PacBio Template Preparation and Sequencing Guide (Pacific Biosciences, Menlo Park, CA, USA) and sequenced by the single-molecule real-time.

The (SMRT) technique was completed using an RS II instrument (Pacific Biosciences) at the McGill University and Genome Quebec Innovation Centre. DNA was also extracted using a KingFisher/Qiagen blood kit and prepared for Illumina MiSeq sequencing using a Nextera XT kit. The genome was first assembled de novo using the Hierarchical Genome Assembly process (HGAP) [18], and the Illumina data were used to correct and validate the entire sequence; the only errors encountered in the PacBio data were homopolymer undercounts. Chromosomes were automatically annotated using an in-house method based on Prodigal [19]; plasmids were manually annotated using Artemis [20] and Blastp on NCBI (https://blast.ncbi.nih.gov/Blast.cgi accessed on 1 March 2021) ANI’s were calculated using OrthoAnIu from EZBioCloud [21]: (https://www.ezbiocloud.net/tools/ani, accessed on 1 July 2021). Plasmid incompatibility groups were determined using PlasmidFinder [22] at the site of the Center for Genomic Epidemiology: (https://cge.dtu.dk/services/PlasmidFinder, accessed on 1 June 2021).

## Data Availability

The chromosomal and plasmid sequences are available in GenBank, accession numbers: *K. michiganensis* CP081351-CP081353; *C. farmeri* CP081314-CP081318.
